# Astroglial Ca^2+^-Dependent Hyperexcitability Requires P2Y_1_ Purinergic Receptors and Pannexin-1 Channel Activation in a Chronic Model of Epilepsy

**DOI:** 10.3389/fncel.2018.00446

**Published:** 2018-11-23

**Authors:** Mario Wellmann, Carla Álvarez-Ferradas, Carola J. Maturana, Juan C. Sáez, Christian Bonansco

**Affiliations:** ^1^Centro de Neurobiología y Plasticidad Cerebral CNPC, Instituto de Fisiología, Facultad de Ciencias, Universidad de Valparaíso, Valparaíso, Chile; ^2^Escuela de Fonoaudiología, Facultad de Medicina, Universidad de Valparaíso, Valparaíso, Chile; ^3^Escuela de Ciencias de la Salud, Universidad Viña del Mar, Valparaíso, Chile; ^4^Departamento de Ciencias Fisiológicas, Facultad Ciencias Biológicas, Pontificia Universidad Católica de Chile, Santiago, Chile; ^5^Instituto de Neurociencias, Centro Interdisciplinario de Neurociencias de Valparaíso, Universidad de Valparaíso, Valparaíso, Chile

**Keywords:** kindling, gliotransmission, astrocyte-to-astrocyte signaling, hemichannels, purinergic receptors, epilepsy

## Abstract

Astrocytes from the hippocampus of chronic epileptic rats exhibit an abnormal pattern of intracellular calcium oscillations, characterized by an augmented frequency of long lasting spontaneous Ca^2+^ transients, which are sensitive to purinergic receptor antagonists but resistant to tetrodotoxin. The above suggests that alterations in astroglial Ca^2+^-dependent excitability observed in the epileptic tissue could arise from changes in astrocyte-to-astrocyte signaling, which is mainly mediated by purines in physiological and pathological conditions. In spite of that, how purinergic signaling contributes to astrocyte dysfunction in epilepsy remains unclear. Here, we assessed the possible contribution of P2Y_1_R as well as pannexin1 and connexin43 hemichannels—both candidates for non-vesicular ATP-release—by performing astroglial Ca^2+^ imaging and dye uptake experiments in hippocampal slices from control and fully kindled rats. P2Y_1_R blockade with MRS2179 decreased the mean duration of astroglial Ca^2+^ oscillations by reducing the frequency of slow Ca^2+^ transients, and thereby restoring the balance between slow (ST) and fast transients (FT) in the kindled group. The potential contribution of astroglial pannexin1 and connexin43 hemichannels as pathways for purine release (e.g., ATP) was assessed through dye uptake experiments. Astrocytes from kindled hippocampi exhibit three-fold more EtBr uptake than controls, whereby pannexin1 hemichannels (Panx1 HCs) accounts for almost all dye uptake with only a slight contribution from connexin43 hemichannels (Cx43 HCs). Confirming its functional involvement, Panx1 HCs inhibition decreased the mean duration of astroglial Ca^2+^ transients and the frequency of slow oscillations in kindled slices, but had no noticeable effects on the control group. As expected, Cx43 HCs blockade did not have any effects over the mean duration of astroglial Ca^2+^ oscillations. These findings suggest that P2Y_1_R and Panx1 HCs play a pivotal role in astroglial pathophysiology, which would explain the upregulation of glutamatergic neurotransmission in the epileptic brain and thus represents a new potential pharmacological target for the treatment of drug-refractory epilepsy.

## Introduction

Under physiological conditions, besides providing metabolic support for neural tissue, astrocytes are essential for neural function and actively participate in the modulation of neuronal excitability and synaptic plasticity (Fellin et al., 2007; Perea and Araque, 2007; Shigetomi et al., 2008; Bonansco et al., 2011; Tan et al., 2017). Endowed with Ca^2+^-dependent excitability, astrocytes can reciprocally communicate among themselves as well as with neurons via gliotransmitters, including glutamate and ATP (Hamilton and Attwell, 2010; Torres et al., 2012; Sahlender et al., 2014). It has been suggested that paracrine astrocyte-to-astrocyte signaling employs ATP as its main extracellular messenger for the propagation of intercellular Ca^2+^ signals, activating P2Y receptors (P2YR) in neighbor cells (Guthrie et al., 1999; Anderson et al., 2004; Bennett et al., 2006; Bowser and Khakh, 2007). Besides vesicular Ca^2+^-dependent exocytosis (Lalo et al., 2014; Lee et al., 2015; Kinoshita et al., 2018), previous works indicate that ATP can reach the extracellular space by means of non-exocytotic mechanisms such as diffusion via poorly selective membrane channels including hemichannels formed by connexin43 or pannexin1 (Cx43 HCs and Panx1 HCs, respectively; Cotrina et al., 1998; Iwabuchi and Kawahara, 2011; Orellana et al., 2011, 2013; Suadicani et al., 2012; Beckel et al., 2014; Chever et al., 2014), nevertheless, their physiological contribution is still a matter of intense debate (Cheung et al., 2014; Harada et al., 2016; Orellana, 2016).

In several neurological diseases, including epilepsy, astrocytes change their gene expression patterns and consequently their morphology, taking up a characteristic phenotype known as reactive astrogliosis (Wetherington et al., 2008; Burda and Sofroniew, 2014). In the epileptic brain, reactive astrocytes exhibit severe changes in the expression of a wide variety of proteins regulating purinergic signaling as well as extracellular concentration of ATP and its metabolites, including over-expression of Cx43, Panx1, P2 receptors as well as ectonucleotidases and adenosine kinases (Bonan et al., 2000a,b; Gouder et al., 2004; Fedele et al., 2005; Aronica et al., 2011; Shen et al., 2014; Barros-Barbosa et al., 2016; Cieślak et al., 2017). Indeed, recent evidence suggests that changes in glial purinergic signaling could be specifically associated with the physiopathology of epilepsy and seizure generation (Ding et al., 2007; Santiago et al., 2011; Pascual et al., 2012; Alves et al., 2017). However, the contributions of astroglial ATP to the epileptogenesis process and the generation of epileptiform activity remain unclear (Cieślak et al., 2017).

We previously showed that astrocytes from the epileptic hippocampus display an abnormal pattern of spontaneous intracellular Ca^2+^-elevations characterized by somatic transients of longer duration (slow transients, STs), which were action-potential independent (i.e., TTX resistant) and sensitive to purinergic antagonists (Álvarez-Ferradas et al., 2015). This astroglial Ca^2+^-mediated hyperexcitability is known to up-regulate glutamate-mediated gliotransmission, increasing basal excitatory neurotransmission in CA3-CA1 synapses by means of a presynaptic mechanism. Since astroglial STs described in this model were highly TTX-insensitive, it is highly likely that astroglial hyperexcitability could be the result from an alteration in astrocyte-astrocyte signaling itself. However, whether ATP-mediated astrocyte-astrocyte signaling contributes to astroglial dysfunction in this chronic model of hippocampal epilepsy is still unknown.

By using a chronic epilepsy model, we show here that hyperexcitability observed in hippocampal astrocytes is driven by purinergic signaling, likely ATP, and requires the participation of Panx1 HCs and subsequent P2Y_1_R activation. Thus, Panx1 HCs and P2Y_1_Rs expressed in astrocytes could represent a novel therapeutic target for seizure control and epilepsy treatment.

## Materials and Methods

All protocols were performed in accordance to the Care and Use of Laboratory Animals National Research Council of the National Academy of Science guidelines, as administered by the Comité Institucional de Bioética para la Investigación en Animales de Experimentación at the Universidad de Valparaíso (CIBICA-UV, Animal Protection Law 20380, Chile). All procedures for experimental handling and sacrificing animals were approved by the CIBICA-UV committee.

### Stereotaxic Surgery and Kindling Protocol

Male Sprague-Dawley rats (at 35 postnatal days, p35) were anesthetized and subjected to stereotactic surgery for electrode implantation (Greenwood et al., 1991; Corcoran et al., 2011). Five electrodes were implanted: one in the right basolateral amygdala complex for stimulation and recording; and two bilaterally implanted pairs for recording in the primary motor (Br. −2.12 mm, ML. ±2.0 mm, DV. 1.5 mm) and the visual cortex (Br. −6.12 mm, ML. ±2.5 mm, DV. 1.5). Coordinates for electrodes implantation were obtained from stereotaxic atlas of Paxinos and Watson (1998). Following surgical procedures, rats had at least 1 week for recovery before to start the kindling protocol.

To induce epileptogenesis, we employed a rapid kindling protocol (RK) developed in our laboratory (Morales et al., 2014; Álvarez-Ferradas et al., 2015). In brief, 10 daily trains of biphasic rectangular current pulses at subthreshold after-discharge (AD) intensity were applied, for 3 days. Rats subjected to RK protocol display progressive epileptic activity throughout the brain accompanied by seizures that increase in severity as the protocol goes on (Morales et al., 2014). The ADs progression were assessed using EEG recordings while seizures were assessed using the Racine scale (Racine, 1972). Rats were considered as epileptic (fully kindled) after having suffered at least five consecutive generalized epileptic seizures (i.e., Racine 4 and 5 states) accompanied by long-lasting repetitive ADs. Under these conditions, cortical and mesolimbic structures of fully kindled rats are considered epileptic tissue, including the hippocampal formation (Shi et al., 2007; Morales et al., 2014; Álvarez-Ferradas et al., 2015).

The control group consisted of rats subjected to electrode implantation surgery but remained non-stimulated (i.e., sham) and naïve animals; results were pooled together because no significant differences were found in previous reports (Álvarez-Ferradas et al., 2015). All the experiments were performed at most 1 week after reaching the fully kindled state, remaining *ad libitum* before, during and after all procedures.

### Hippocampal Slice Preparation

Acute hippocampal slices were obtained from control and kindled rats as previously described (Bonansco et al., 2002; Fuenzalida et al., 2010). Briefly, rats were anesthetized and decapitated, the brain was rapidly removed through craniotomy and placed in ice-cold (<4°C) and sucrose-enriched artificial cerebrospinal fluid (ACSF) containing (in mM): Sucrose 215.0, 2.5 KCl, glucose 20.0, 26.0 NaHCO_3_, 1.6 NaH_2_PO_4_, 1 CaCl_2_, 4 MgCl and 4 MgSO_4_ gassed with 95% O2/5% CO_2_ (pH = 7.4). Transversal brain slices (300–350 μm) were obtained by using a Vibroslice microtome (VSL, WPI, USA) and then incubated in regular ACSF solution for 1 h at room temperature (21–24°C), containing (in mM): 124.0 NaCl, 2.7 KCl, 1.25 KH_2_PO_4_, 2.0 Mg_2_SO_4_, 26.0 NaHCO_3_, 2.5 CaCl_2_ y 10.0 glucose. Slices were then transferred to an immersion recording chamber and superfused with carbogen-bubbled ACSF (2 ml/min).

### Ca^2+^ Imaging in Astrocytes

**Figure 1:**
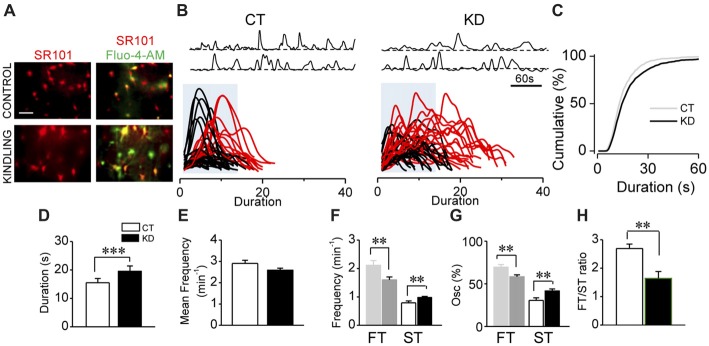
Kindled hippocampal slices display spontaneous astroglial Ca^2+^-mediated hyperexcitability. **(A)** Representative fluorescence microscopy images of SR101^+^ astrocytes (red; left) and maximum intensity projection obtained from 5 min video of fluorescence transients for Fluo-4-AM- loaded astrocytes of CA1 area (stratum radiatum) showed colocalization (yellow; right) both in control and kindled hippocampal slices (calibration bar 30 μm). **(B)** Representative Ca^2+^-fluorescence traces of spontaneous Ca^2+^ elevations in two astrocytes (top). Superimposition of spontaneous Ca^2+^ elevations obtained from several astrocytes (>10 cells), aligned at the start of each single transient. Some slow Ca^2+^ transients (>50 oscillations) were highlighted in red; blue boxes show the temporal window within 17 s (fast transients [FTs]; slow transients [STs] surpasses the blue box), detected in control (left, CT) and kindled (right, KD) slices. **(C)** Cumulative distribution plot of the spontaneous Ca^2+^ events duration obtained from all astrocytes analyzed for CT (*n* = 41; left) and KD hippocampi (*n* = 76; middle). Summary graphs of astroglial Ca^2+^ oscillations mean duration **(D)**, mean frequency **(E)**, ST and FT frequency **(F)**, ST and FT oscillation percentage **(G)** and FT/ST ratio **(H)** for control and kindled groups. Statistics: Shapiro-Wilk test was employed for the distribution analysis of Ca^2+^ signals. Mann-Whitney-test and *T*-test were used for single comparisons and ANOVA with Bonferroni *post hoc* test for multiple comparisons (*p < 0.05; **p < 0.01; ***p < 0.001).

Spontaneous astroglial Ca^2+^-mediated activity was monitored by fluorescence microscopy using Fluo4-AM as a Ca^2+^ indicator. Slices were incubated first with the astroglial morphological marker sulforhodamine-101 (SR101; 0.5–1 μM; Kafitz et al., 2008) for 20–30 min in low Ca^2+^/high Mg^2+^ ACSF at 32–34°C in order to confirm the specific recording of Ca^2+^ signals from astrocytes. Slices were then transferred to a maintenance chamber with regular ACSF for 30 min and later incubated with FLUO4-AM (1–2 μL of the dye with pluronic acid at 0.01% was dropped over the hippocampus, obtaining a final concentration of 5–10 μM) for 60–75 min in regular ACSF at room temperature. Under these conditions, most of loaded cells with Fluo4-AM are astrocytes (Figure 1A; Aguado et al., 2002; Navarrete and Araque, 2008). The following modifications were made to our previous incubation protocol (Álvarez-Ferradas et al., 2015) in order to improve the overall quality and the noise-to-signal ratio of the fluorescence recordings: (i) substitution of regular ACSF for sucrose-enriched ACSF for brain extraction and slice preparation (215.0 mM); (ii) longer incubation period with FLUO-4-AM (from 30 to 60 min to 60–75 min); and (iii) longer incubation period in regular ACSF after FLUO-4-AM incubation (from 15 to 30 min to 30–40 min). Astrocytes were imaged using a CCD camera (Andor DR328G; Andor Technologies plc, Ireland) attached to a fluorescence microscope (Nikon, Japan). The camera was controlled and synchronized by the Niss-Elements AR 3.2 software (Nikon, Japan), that was also used for control and offline analysis. Cells were illuminated with a xenon lamp at 490 nm (200–400 ms exposure; 36,700 mm^2^ area), and images were acquired at 1 Hz for 5 min, regulated by a shutter (Lambda SC-Smart shutter, Sutter Instrument Company). Analysis of astrocyte Ca^2+^ levels was restricted to the cell body and Ca^2+^ transients were estimated as changes in the fluorescence signal over the baseline (ΔF/F0) after background subtraction. For every individual cell, a baseline was obtained by extracting the fluorescence values of at least 30 consecutive frames were the astrocyte exhibited no spontaneous Ca^2+^-dependent activity. Two values were obtained from the baseline: an average of the basal fluorescence levels and the standard deviation. Changes in fluorescence were considered as events and automatically picked up by the analysis software when the ΔF/F0 intensity exceeds the fluorescence of the baseline in three or more standard deviations (i.e., trigger), at least for five consecutive frames. For multi-peak astroglial Ca^2+^ activity, those events where the ΔF/F0 dropped to half of the maximum fluorescence intensity in relation to the baseline were considered as independent transients.

### Dye Uptake, Immunofluorescence and Confocal Microscopy

All the procedures and reagents for dye uptake, immunofluorescence and confocal microscopy imaging were described previously (Maturana et al., 2016). In all the experiments, hippocampal slices from control and kindled rats were processed in parallel. In brief, acute hippocampal slices from control and kindled rats were treated for 20 min with antagonist or mimetic peptides (MRS2179, ^10^Panx, ^10^Panx SCR, GAP26, GAP26 SCR) at room temperature in regular carbogen-bubbled ASCF and then incubated with 4 μM ethidium bromide (EtBr) for 10 min. Afterwards, slices were washed three times for 5 min in regular ACSF and fixed for 1 h in 4% paraformaldehyde at 48°C. To quantify dye uptake in a particular cell type, slices were then processed for immunofluorescence detection of a specific molecular marker for the cell type of interest (see below). For immunofluorescence and confocal microscopy imaging, fixed brain slices were incubated for 1 h in blocking solution (PBS containing 0.2% gelatin and 1% Triton X-100) and then incubated overnight at 48°C with the following primary antibodies diluted in blocking solution containing 10% regular donkey serum. The primary antibodies were: Monoclonal mouse anti-GFAP antibody (1:300) for astrocytes, polyclonal rabbit anti-Iba1 antibody (1:100) for microglia and monoclonal mouse anti-NeuN antibody (1:300) for neurons. Afterwards, samples were washed five times with PBS and incubated at room temperate with donkey anti-mouse Alexa Fluor 488 (1:500), donkey anti-rabbit Alexa Fluor 488 (1:500), donkey anti-goat Cy2 (1:300), donkey anti-mouse Cy3 (1:500), donkey anti-rabbit Cy3 (1:500), donkey anti-goat TRITC (1:100), or donkey anti-chicken Cy3 (1:300) antibodies. Samples were washed and mounted with DAPI-flouromount-G and images were taken with a confocal microscope Eclipse Ti-E Nikon. Stacks of consecutive images were taken with a 60× objective at 250 nm interval with three lasers (405, 488 and 561 nm), and Z projections were reconstructed with Leica software. Levels of dye uptake in control and kindled rats were compared as fluorescence intensity, which was digitized in arbitrary units in 65,000 shades of gray using ImageJ software and corrected total cell fluorescence was calculated (Chever et al., 2014; Orellana et al., 2015). The corrected total cell fluorescence is represented as relative fluorescence intensity (RFI) = integrated density − (area of selected cell × mean fluorescence of background readings). An outline was drawn around each cell and circularity, area and mean fluorescence were measured, along with several adjacent background readings. The total number (*n*) of cells counted for each group was approximately 60. At least three fields were selected in every slice.

### Reagents

Membrane-permeable fluorescent calcium indicator FLUO4-AM and SR101 were purchased from Molecular Probes (USA). Chemicals were purchased from Sigma-Aldrich (USA) and Tocris (UK). The specific antagonist of P2Y1Rs, 2’-Deoxy-N6-methyladenosine 3’,5’ bisphosphate tetrasodium salt (MRS2179; 10 μM) was added directly to the perfusion system. For Cx43 and Panx1 hemichannel blockade in Ca^2+^imaging experiments, slices were previously treated with ^10^Panx (WRQAAFVDSY) or GAP26 (VCYDKSFPISHVR; both 200 μM, diluted in regular ASCF) for 30 min at room temperature and then imaged. Mimetic peptides were purchased from SBSBIO (Beijin, China).

### Statistical Analysis

In all cases, the distribution of each population was first determined (Shapiro-Wilk test, Kolmogorov-Smirnov test) before applying statistical comparisons, which were made using parametric Student’s *t*-test, Mann-Whitney Test as deemed appropriate. For multiple comparisons ANOVA or Kruskal-Wallis test was used as appropriate, both with *post hoc* Bonferroni correction. Data are expressed as the mean plus standard error (SEM). Differences were considered statistically significant when *p* < 0.05 (*); *p* < 0.01 (**) or *p* < 0.001 (***), as indicated. Blind experiments were not performed but the same criteria were applied to all groups for comparisons. Randomization was not employed.

## Results

### Astroglial Hyperexcitability in the Kindled Hippocampus

In order to assess the parameter for astroglial hyperexcitability using the modified incubation protocol (see “Materials and Methods” section), we analyzed spontaneous intracellular Ca^2+^ transients from control and kindled hippocampal slices pre-incubated with the astrocyte-specific marker SR101 and the Ca^2+^ probe Fluo-4-AM (Figure 1A). The modifications introduced in the incubation protocol (see “Materials and Methods” section) increased by 100% both the number of astrocytes exhibiting Ca^2+^ transients and the number of Ca^2+^ transients per cell. According to our previous findings (Álvarez-Ferradas et al., 2015), the average duration of Ca^2+^ transients in the KD group remained higher than in the CT group, but slightly lower in both groups compared to the values previously reported (Figures 1B–D; CT 15.5 ± 1.4 s, median 13 s, *n* = 41; KD 19.6 ± 1.8 s, median 15 s, *n* = 76; *p* = 0.001), whereas mean frequencies showed no differences between groups (Figure 1E; *p* = 0.062). The duration of Ca^2+^ transients was distributed among two populations from the 75th percentile of cumulative distribution, corresponding to 17 s and utilized as a cut-off criterion, from which the random events in a same astrocyte were classified into slow (STs >17 s) or fast transients (FTs <17 s; Figure 1B, lower). The aforementioned was confirmed through the comparison of the two data sets using Kolmogorov-Smirnov test (*p* < 0.0001, *D* = 0.1155), with the maximum difference between populations above the 75th percentile. Although FTs were the most prevalent events in both groups, ST frequency in KD was higher than in CT (Figure 1F; CT 0.79 ± 0.06 min^−1^; KD 0.98 ± 0.03 min^−1^; *p* = 0.04), while FT frequency was lower in the KD group (Figure 1F; CT 2.11 ± 0.16 min^−1^; KD 1. 61 ± 0.09 min^−1^, *p* = 0.01). Additionally, 41.70 ± 2.25% of the oscillations corresponded to ST, whereas only 30.50 ± 3.03% was observed in the control groups (Figure 1G, *p* = 0.027). Since these differences in FT and ST frequencies between groups could be attributed to a disruption of astroglial excitability, we incorporated the FT/ST frequency ratio as an index of astrocytic hyperexcitability, where a lower index would be associated to greater Ca^2+^-dependent excitability. In epileptic rats, the FT/ST index value was significantly lower than in the CT group (Figure 1H; 1.67 ± 0.24 vs. 2.69 ± 0.46, respectively; *p* = 0.035). These findings confirm our previous evidence, and indicate that astrocytes from the epileptic hippocampus exhibit increases in Ca^2+^-dependent excitability characterized by a higher occurrence of long-lasting transients, expressed as a lower FT/ST frequency ratio.

### Astroglial Slow Ca^2+^ Transients Require P2Y_1_R Activation

**Figure 2:**
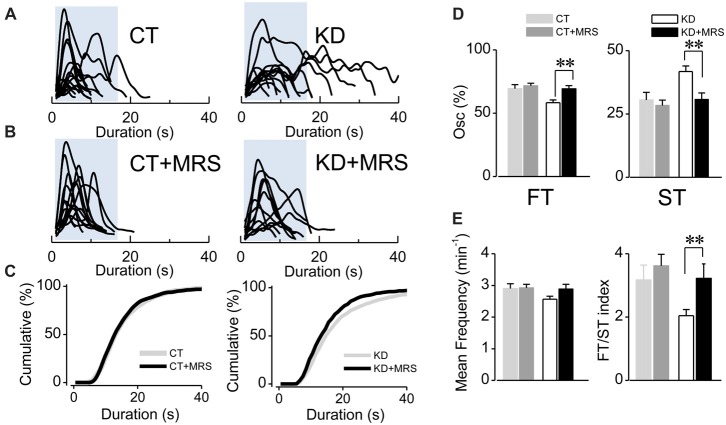
Ca^2+^-mediated astroglial hyperexcitability requires P2Y_1_R activation. **(A)** Aligned traces of multiple Ca^2+^ oscillations from two representative CT and KD astrocytes recorded in basal conditions (ACSF medium) and **(B)** 20 min after the addition of the selective P2Y_1_R antagonist MRS2179 (10 μM, MRS) in the perfusion system. **(C)** Cumulative distribution plots of the spontaneous Ca^2+^ events before and after the application MRS. **(D)** Percentage of FTs (left) and STs (right) per astrocyte before and after the treatment with the antagonist. **(E)** Summary graphs of the mean frequency (left) and FT/ST index (right) obtained before and 20 min after the addition of MRS from both CT and KD groups. Notice that MRS affects astrocyte Ca^2+^-dependent activity only in the KD group, but did not modify any parameter of the Ca^2+^ signals measured in the CT group. Statistics: Shapiro-Wilk test was employed for the distribution analysis of astrocytic Ca^2+^ signals. Mann-Whitney test and paired *T*-test were used for single comparisons; ANOVA with Bonferroni *post hoc* analysis for multiple comparisons (*p < 0.05; **p < 0.01; ***p < 0.001).

Since astroglial slow somatic Ca^2+^ transients in the epileptic hippocampus are independent from neuronal activity (i.e., TTX-insensitive) and sensitive to purinergic antagonists for P2Y_1_R (Álvarez-Ferradas et al., 2015), the imbalance between FT and ST expressed as a lower FT/ST index could have resulted from a disruption of astrocyte-to-astrocyte signaling mediated by ATP. To assess this idea, we evaluated whether P2Y_1_R activation is specifically required for the generation of slow Ca^2+^ transients, or for both ST and FT. To this end, astroglial fluorescence signals were recorded in the presence of the specific P2Y_1_R antagonist MRS2179 (MRS; 10 μM). If P2Y_1_R activation is required to generate both ST and FT, the receptor blockade would diminish the frequency and percentage of both types of oscillations, without changing the FT/ST index. Conversely, if P2Y_1_R activation is implicated in the higher incidence of ST in the epileptic hippocampus, the antagonist would diminish only frequency and percentage of ST, reaching an FT/ST index similar to that exhibited by the control group. In fact, the average duration of Ca^2+^ transients diminished in the presence of MRS in kindled rats (Figures 2A–C; KD 19.57 ± 1.85 s, median 15 s, *n* = 76; KD plus MRS 15.61 ± 1.38 s, median 13 s, *n* = 49, *p* < 0.0001). This decrease is a consequence of a drop in ST percentage (Figure 2D, KD 41.74 ± 2.25%, median 38.4%; KD plus MRS 30.72 ± 2.57%, median 25%; *p* = 0.007), accompanied by an increase in FT percentage (Figure 2D; KD 58.25 ± 2.25%, median 61.53%; KD plus MRS 69.27 ± 2.57%, median 75%). Consequently, the FT/ST index in kindled rats increased more than 50%, reaching the values observed in CT (Figure 2E; KD 2.04 ± 0.19; KD plus MRS 3.22 ± 0.45; *p* = 0.037). Interestingly, P2Y_1_R blockade in the control group did not produce changes in average transient duration (Figures 2B,C; *p* = 0.47), the percentage of ST (Figure 2D; *p* = 0.72) and the FT/ST index (Figure 2E; *p* = 0.79). Moreover, MRS application did not change the mean frequency in both CT and KD conditions (CT 2.90 ± 0.15 min^−1^; CT plus MRS 2.92 ± 0.11 min^−1^; KD 2.56 ± 0.09 min^−1^; KD plus MRS 2.88 ± 0.15 min^−1^; *p* = 0.076), suggesting that P2Y_1_R blockade restored the balance between FT and ST without modifying the total amount of astroglial activity. Taken together, these results suggest that slow astroglial Ca^2+^ transients require P2Y_1_R activation, which is responsible for the FT/ST imbalance that characterizes abnormal excitability shown by astrocytes from the epileptic brain.

### Enhanced Activity of Panx1 HCs in Astrocytes From the Kindled Hippocampus

Astroglial ATP can reach the extracellular medium through different mechanisms, including Ca^2+^-dependent exocytosis, hemichannels and ligand-gated receptor channels (Sahlender et al., 2014; Harada et al., 2016). One of the main candidates for ATP release are Panx1 HCs and Cx43 HCs, whose activity and expression has been mostly founded in pathological conditions, including epilepsy (Mylvaganam et al., 2014). Therefore, by employing specific blockers, we next assessed whether astroglial Panx1 and Cx43 HCs are functional in basal conditions measuring EtdBr uptake in acute slices from kindled and control rats.

**Figure 3:**
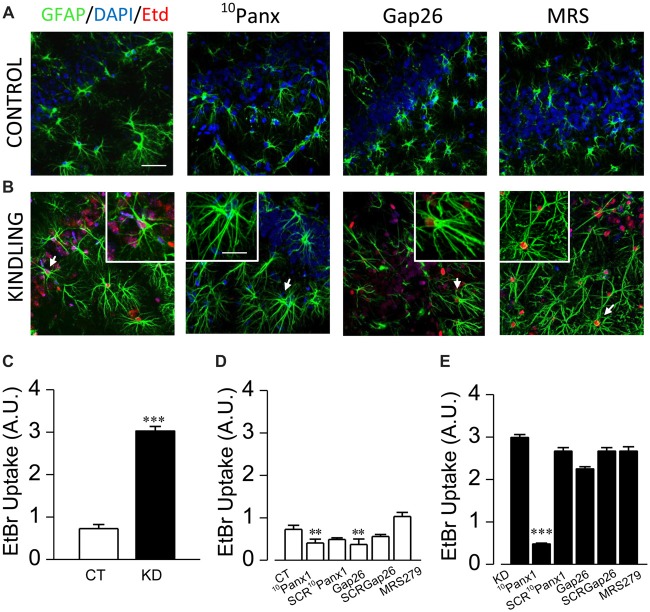
Panx1 HCs activity is enhanced in astrocytes from kindled hippocampi. Representative EtBr uptake (red) in stratum radiatum and nuclear layer (blue, DAPI) astrocytes labeled with GFAP (green) from control **(A)** and kindled **(B)** hippocampal slices taken in basal conditions (left end) and in the presence of ^10^Panx1 (200 μM), Gap26 (200 μM) and MRS2179 (MRS, 10 μM), applied 30 min prior EtBr uptake assays (scale bar, 25 μm). Astrocytes magnified in each image insert are indicated with white arrows (scale bar, 10 μm). **(C)** Mean values of EtBr uptake, expressed in arbitrary units, measured from CT and KD slices in basal conditions. Mean astrocytic EtBr uptake in basal conditions and in the presence of ^10^Panx1 (200 μM), ^10^Panx1 scrambled (SCR^10^Panx1 200 μM), Gap26 (200 μM), Gap26 scrambled (SCRGap26, 200 μM) and MRS (10 μM) for CT **(D)** and **(E)** KD groups. All averaged data was obtained from *n* = 100 cells and six slices for each condition; *T*-test for single comparisons and ANOVA with Bonferroni *post hoc* analysis for multiple comparisons (*p < 0.05; **p < 0.01; ***p < 0.001).

According with previous descriptions made in our epilepsy animal model (Morales et al., 2014), astrogliosis was confirmed in all hippocampal formation of kindled rats, with a great number of GFAP^+^ astrocytes (KD 20.3 ± 1.0 cells/mm^2^, *n* = 11 slices; CT 13.9 ± 0.7 cells/mm^2^, *n* = 11 slices; *p* < 0.001), which showed thicker, elongated and more sinuous processes located in both stratum radiatum and oriens, as well as in pyramidal layers. In basal conditions, reactive astrocytes showed three times higher EtBr uptake compared to the control group (Figure 3C; KD 2.98 ± 0.08; CT 0.95 ± 0.05; *p* < 0.0001). In order to test the contribution of Panx1 and Cx43 HCs in EtBr membrane permeation, we employed the mimetic peptides ^10^Panx1 and Gap26, which are selective blockers for Panx1 and Cx43 HCs, respectively (Giaume et al., 2013). In the control group, both ^10^Panx1 and Gap26 diminished basal EtBr uptake in GFAP^+^ astrocytes (Figure 3D; ^10^Panx1 0.30 ± 0.04, *p* = 0.0031; Gap26 0.58 ± 0.06, *p* = 0.0226). In KD slices, ^10^Panx1 blocked up to 85% of basal EtBr uptake in reactive astrocytes, while the effect of Gap26 reached just 20% (Figure 3E; ^10^Panx1 0.48 ± 0.03, *p* < 0.0001; Gap26 2.42 ± 0.06; *p* = 0.331). Since there is evidence suggesting a modulatory effect of P2 receptors on the gating of Panx1 HC (Dubyak, 2009), we assessed whether P2Y1Rs could have an acute effect on Panx1 gating in reactive astrocytes. In the presence of MRS, basal EtBr uptake did not significantly change in both kindled and control astrocytes (Figure 3E; *p* = 0.52). The lack of acute effect of MRS in our experimental conditions suggests that the increased Panx1 HC activity in reactive astrocytes is not regulated by G-coupled P2 receptors or the changes in astroglial Ca^2+^-dependent activity induced by their activation.

### Astrocytic Slow Ca^2+^ Transients Require Panx1 HC Activation in the Kindled Hippocampus

**Figure 4:**
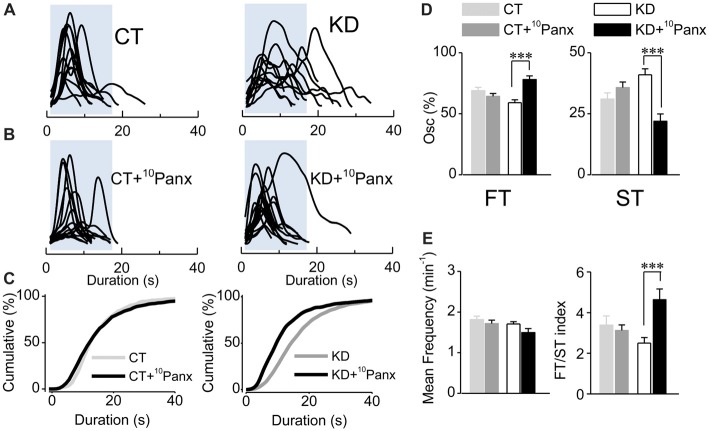
Purinergic dependent astroglial ST requires Panx1 HC activity. **(A)** Aligned traces of multiple Ca^2+^ oscillations recorded in basal conditions (ACSF medium) from two representative CT and KD astrocytes. **(B)** Aligned traces of multiple Ca^2+^ oscillations recorded from two astrocytes belonging to hippocampal slices pre-incubated with the mimetic peptide ^10^Panx1 (200 μM; >30 min). **(C)** Cumulative distribution plots of the spontaneous Ca^2+^ events in basal conditions and in slices incubated with ^10^Panx1 for both groups. **(D)** Percentage of FTs (left) and STs (right) per astrocyte in basal conditions and in the presence of ^10^Panx1. **(E)** Summary graphs of the mean frequency (left) and FT/ST index (right) obtained in ASCF and ^10^Panx1 treated slices from CT and KD groups. Notice that the inhibition of Panx1 HC affected astrocyte Ca^2+^-dependent activity only in the KD group, but did not modify any parameter of the Ca^2+^ signals measured in the CT group. Statistics: Shapiro-Wilk test was employed for the distribution analysis of astrocytic Ca^2+^ signals. Mann-Whitney test and paired *T*-test were used for single comparisons; ANOVA with Bonferroni *post hoc* analysis for multiple comparisons (*p < 0.05; **p < 0.01; ***p < 0.001).

Since EtBr uptake in KD astrocytes was mostly blocked by ^10^Panx1-mimetic peptides, we next asked whether astroglial hyperexcitability requires Panx1 HC activity. Slices from control and kindled rats were pre-incubated with ^10^Panx1 (200 μM; >30 min), and then spontaneous astroglial Ca^2+^-signaling was recorded. In the kindling group, Panx1 HC blockade induced a decrease in the average duration of astrocytic Ca^2+^ transients (Figures 4A–C; KD 18.60 ± 1.34 s, median 15 s, *n* = 127; KD plus ^10^Panx1 13.56 ± 1.83 s, median 10 s, *n* = 75; *p* < 0.0001). Furthermore, the total percentage of the ST component decreased (Figure 4D, KD 40.97 ± 2.4%, median 38.46, KD plus ^10^Panx1 21.94 ± 2.99%, median 14.28%) proportionally to the increase of the FT component (Figure 4D; KD 59.02 ± 2.4%, median 61.53%, KD plus ^10^Panx1 78.05 ± 2.99%, median 85.71%; *p* < 0.0001). ^10^Panx1 reduced ST frequency without changing FT frequency, increasing the FT/ST index to values similar to those observed in the control group (Figure 4E; KD 2.49 ± 0.27; KD plus ^10^Panx1 4.63 ± 0.52; *p* < 0.0001; CT 3.38 ± 0.44; CT plus ^10^Panx 3.12 ± 0.26; *p* = 0.05). In the CT group, ^10^Panx1 had no effect on the average duration of astroglial Ca^2+^ oscillations (Figures 4A–C; *p* = 0.3) or the percentage of FT and ST (Figure 4D; *p* = 0.32) and hence, FT/ST index remained without changes (Figure 4E; *p* = 0.86). Notably, Panx1 HC blockade did not produce changes in mean frequency in both groups (Figure 4E; *p* = 0.058), indicating that the effect of ^10^Panx1 is restricted to the modulation of the ST component, without affecting the overall activity of the astrocytes. Taken together, these results strongly suggest that the astroglial hyperexcitability observed in the kindled hippocampus require the activation of Panx1 HCs, which likely acts as the release mechanism for the purinergic mediator that activates P2Y1R and increases the duration of Ca^2+^ oscillations.

## Discussion

The above results indicate that the abnormal pattern of astroglial Ca^2+^-mediated signals observed in the hippocampus of chronic epileptic rats is likely driven by purinergic astrocyte-astrocyte signaling, and requires the activity of Panx1 HCs and P2Y_1_R. Panx1 HCs probably acts as the pathway through which purines reach the extracellular medium, which activates P2Y_1_R and triggers an abnormal pattern of astroglial Ca^2+^-mediated activity. Those mechanisms could cause an increase in the release of glutamate from astrocytes, which in turn upregulates glutamatergic neurotransmission by modulating the presynaptic component in the epileptic hippocampus (Álvarez-Ferradas et al., 2015), explaining the increased glutamatergic tone in the epileptic brain.

### Astroglial Ca^2+^-Dependent Hyperexcitability in Epileptic Rats Requires P2Y_1_R Activation

Although glial purinergic receptors have been implicated in the generation and spread of epileptiform activity during seizures (Kumaria et al., 2008; Engel et al., 2012, 2016; Alves et al., 2017), their specific contributions and mechanisms through which they participate in generating epileptiform activity and epileptogenesis were unknown. Here, we show that the P2Y_1_R antagonist MRS2179 normalize the duration of astroglial Ca^2+^ oscillations in the kindled group by reducing the frequency and percentage of STs, but with no effect either in the duration or the frequency of Ca^2+^ transients in the control condition (Figure 2). Previously, we showed that astroglial STs in KD hippocampus are insensitive to TTX, whereas are completely abolished in the control group by this toxin (Álvarez-Ferradas et al., 2015). The duration of spontaneous somatic astroglial Ca^2+^ transients reported here are in accordance with those previously described which ranged from 5 s to 200 s (Parri et al., 2001; Aguado et al., 2002; Takata and Hirase, 2008; Di Castro et al., 2011; Álvarez-Ferradas et al., 2015). There are few works in which the average duration and median of somatic Ca^2+^ oscillations were reported (Parri et al., 2001; Takata and Hirase, 2008), with great methodological differences between them (i.e., incubation protocol, animal strain and age). Nonetheless, the reported values in these publications are similar to those obtained here. Our new findings suggest that a diffusible purinergic signal released by astrocytes themselves activates astroglial P2Y_1_R (i.e., astrocyte-to-astrocyte signaling) and upregulates the incidence of STs, which is supported by several evidences: (i) TTX-insensitive neurotransmitter release is associated with FTs confined to microdomains in astrocytic processes and not to somatic Ca^2+^ oscillations (Di Castro et al., 2011; Álvarez-Ferradas et al., 2015); (ii) ATP is released by astrocytes as a mechanism for paracrine signaling in several neuropathologies, being P2YR-mediated signaling one of the main pathways for astrocyte-to-astrocyte communication in pathological conditions (Anderson et al., 2004; Iwabuchi and Kawahara, 2011; Pascual et al., 2012; Orellana et al., 2013; Delekate et al., 2014); and (iii) astrocytes generate Ca^2+^ transients as a consequence of P2YR activation (Bowser and Khakh, 2007; Torres et al., 2012), which is also observed in pathological conditions, including epilepsy (Delekate et al., 2014; Álvarez-Ferradas et al., 2015; Ravin et al., 2016; Reichenbach et al., 2018). In addition, it has been shown that P2Y_1_R induces large and long-lasting astrocytic Ca^2+^ transients that do not require action potentials for their generation (Gallagher and Salter, 2003; Shigetomi et al., 2018), which is consistent with our previous findings (Álvarez-Ferradas et al., 2015) and with the decrease in the frequency and percentage of STs associated with P2Y_1_R blockade in the epileptic hippocampus above described (Figure 2). Accordingly, in our experiments P2Y_1_R blockade did not change the mean frequency of Ca^2+^ transients in the epileptic group (Figure 2E), suggesting that P2Y_1_R activation is associated exclusively with the generation of astroglial STs, which is confirmed by the restoration of the FT/ST index. P2Y_1_Rs, preferably expressed in astrocytes (Jourdain et al., 2007; Di Castro et al., 2011), are overexpressed in various neuropathological conditions (Franke et al., 2012; Delekate et al., 2014; Alves et al., 2017). In fact, Alves et al. (2017) showed that astrocytic P2Y_1_Rs are overexpressed in the hippocampus of two other epilepsy models as well as in hippocampal tissue obtained from patients suffering from temporal lobe epilepsy. In the same work, the functional contribution of P2Y_1_Rs was also demonstrated by the central injection of the specific P2Y agonist ADP, which exacerbated seizure severity and duration in mice (Alves et al., 2017).

It should also be noted that ATP-mediated P2Y_1_R activation is one of the key signals for the developing of reactive gliosis in several brain injury conditions (Franke and Illes, 2014; Shinozaki et al., 2017), which could explain the strong astrogliosis described previously in our epilepsy model as well as in others (Pernot et al., 2011; Morales et al., 2014; Buckmaster et al., 2017). Unlike previous reported findings (Di Castro et al., 2011), we observed that spontaneous astroglial somatic Ca^2+^ signals do not require P2Y_1_R activation in the control condition, since their blockade has no effect on Ca^2+^ oscillations (Figure 2). However, we cannot rule out that P2Y_1_R-dependent Ca^2+^ signaling may be occurring at the microdomain level, which is outside our range of microscopic detection. In the same sense, the age of the animals employed in this work may explain this discrepancy, since it is highly likely that astrolgial Ca^2+^ signaling is differentially modulated during the development (Zhu and Kimelberg, 2001; Sun et al., 2013). Also, the region of the CNS (CA1 of the hippocampus) used for the experiments could account for the discrepancy between works, because Ca^2+^ signaling exhibits specific temporal features and dynamics depending on the location of the astrocytes in the CNS (Di Castro et al., 2011; Haustein et al., 2014).

Together, the evidence suggests that astroglial hyperexcitability observed in the hippocampus of kindled rats is likely due to an alteration in astrocyte-to-astrocyte signaling that requires P2Y_1_R activation, being these receptors involved in the generation of astroglial Ca^2+^ ST in epileptic tissue, but not in physiological conditions.

### Panx1 HC Contribution to Astroglial Hyperexcitability

Our results show that EtBr dye uptake in reactive astrocytes from the epileptic group was greater than in control slices, being mediated almost exclusively by Panx1 HCs, and with only a mild contribution from Cx43 HC (Figure 3). Consistent with this, we found that Panx1 HC blockade with the mimetic peptide ^10^Panx1 reduces the percentage and frequency of STs in kindled rats (Figure 4), an effect that was not observed when Cx43 HCs were inhibited with Gap26 (Supplementary Figure S1). Interestingly, Panx1 HCs and Cx43 HC inhibition had no effect on Ca^2+^ signals in astrocytes from the control group (Figure 4; Supplementary Figure S1), suggesting that astroglial STs require Panx1 HCs only in the epileptic condition. In accordance with our results, Panx1 is overexpressed in different cell types from epileptic tissue (Mylvaganam et al., 2010; Jiang et al., 2013), and their blockade with mefloquine reduced seizure severity and duration in kainate-induced status epilepticus (Santiago et al., 2011). Moreover, Panx1-KO mice treated with kainate exhibited less severe seizures and lower extracellular concentrations of ATP compared to kainate-treated WT-mice, strongly suggesting an important role for Panx1 HCs in mediating ATP release in epilepsy (Dubyak, 2009; Santiago et al., 2011; Mylvaganam et al., 2014).

Notably, we observed the same effects on astroglial Ca^2+^ oscillation patterns in P2Y_1_R and Panx1 HC inhibition experiments (Figures 2, 4, respectively), where both specific blockers exclusively reduced the frequency of astroglial STs, and restored FT/ST balance in the kindled group, without affecting the control group. In spite of the above, the exact mechanisms that regulate the opening and release of purines through Panx1 HCs are unknown (Dahl, 2015). Although there is much evidence suggesting that astrocytes express functional Cx43 HCs (Cheung et al., 2014), which also mediate ATP release (Stout et al., 2002; Torres et al., 2012), their role in epilepsy seems to be more closely related to astroglial coupling via gap junctions. Indeed, Cx43/32 gap junction inhibition by carbenoxelone and specific mimetic peptides (e.g., Gap27 and SLS-peptide) diminished recurrent seizure-like activity (Samoilova et al., 2008), and decreased the incidence of epileptiform discharges induced *in vitro* by reducing astrocytic synchronized Ca^2+^-transients (Kékesi et al., 2015). In our conditions, although Gap26 showed only a slight effect on EtBr uptake and no effect at all on astrocytic Ca^2+^ signals (Supplementary Figure S1), our results do not discard the probable contribution of connexin formed gap junctions in astrocyte-to-astrocyte signaling in the epileptic condition. Together, the above evidence strongly suggests that Panx1 HCs are involved in epilepsy pathophysiology, probably mediating the release of astroglial purines (likely ATP) and thereby activating the P2Y1Rs in neighboring astrocytes, being the pathway underlying the astroglial hyperexcitability pattern and upregulating glutamate gliotransmission that increases excitatory neurotransmission in kindled rats (Álvarez-Ferradas et al., 2015). This purine-mediated astrocyte-to-astrocyte pathway could be specifically and exclusively linked to astrocytes in the epileptic tissue, because astrocytes from control rats do not require Panx1 HC or P2Y1R to generate STs. However, further studies must be performed to identify the specific purine, its source, and the signal that triggers the astroglial dysfunction in the epileptic tissue.

It was previously suggested that activated microglia release ATP, which in turn stimulate neighboring astrocytes to release more ATP, amplifying Ca^2+^ waves in the astrocyte network (Pascual et al., 2012). We propose, by means of the results obtained in this work, that astrocytes amplify this signal through Panx1 HCs-mediated ATP release, which activates P2Y1R and triggers the release of more ATP, activating neighbor cells as a loop. This pathway induce long lasting astrocytic Ca^2+^ transients that propagate at a large scale (Gallagher and Salter, 2003; Anderson et al., 2004; Kuchibhotla et al., 2009; Kuga et al., 2011; Shigetomi et al., 2018), up-regulating gliotransmitter release and in turn increasing neuronal excitability in a large territory (Perea and Araque, 2005; Fellin et al., 2007; Shigetomi et al., 2008), as it happens when the epileptiform activity spreads after the activation of the epileptogenic focus (Gómez-Gonzalo et al., 2010; Araque et al., 2014). Thus, astrocyte-to-astrocyte communication could represent a key mechanism in upregulating the glutamatergic tone and promoting the occurrence and recurrence of seizures in the epileptic brain. Also, we provide evidence supporting the idea that reactive astrocytes in the epileptic tissue undergo not only structural changes, but also functional modifications that affect astrocyte-to-astrocyte communication and could alter synaptic transmission and neuronal excitability as a consequence. In that scenario, the disruption of astrocyte-to-astrocyte communication and Ca^2+^-dependent astroglial signaling represents a new functional hallmark of the epileptic tissue and a mechanism likely contributing to the excitatory loop between astrocytes and neurons that drives neurons to seizure threshold.

## Author Contributions

CB conceived and supervised the experiments and wrote the manuscript. MW performed the experiments, analyzed the data and wrote the manuscript. CÁ-F performed the experiments. CM performed EtBr dye uptake experiments and analyzed the data. JS contributed in experimental design of the EtBr uptake experiments and in the edition of the article.

## Conflict of Interest Statement

The authors declare that the research was conducted in the absence of any commercial or financial relationships that could be construed as a potential conflict of interest.
